# Fabrication and Characterization of Eco-Friendly Polyelectrolyte Bilayer Films Based on Chitosan and Different Types of Edible Citrus Pectin

**DOI:** 10.3390/foods11213536

**Published:** 2022-11-07

**Authors:** Xincheng Fu, Xia Chang, Zemin Ding, Haishan Xu, Hui Kong, Fei Chen, Rongrong Wang, Yang Shan, Shenghua Ding

**Affiliations:** 1 Longping Branch, College of Biology, Hunan University, Changsha 410125, China; 2 Hunan Agricultural Product Processing Institute, Hunan Academy of Agricultural Sciences, Hunan Provincial Key Laboratory for Fruits and Vegetables Storage Processing and Quality Safety, Changsha 410125, China; 3 College of Food Science and Technology, Hunan Agricultural University, Changsha 410128, China

**Keywords:** polyelectrolyte bilayer film, eco-friendly, citrus pectin, chitosan, mechanical property, gas barrier, antifogging property

## Abstract

The eco-friendly polyelectrolyte bilayer films were prepared by layer-by-layer (LBL) casting method using chitosan (CS) and four types of edible citrus pectin as film substrates. The results showed that the polyelectrolyte bilayer films exhibited excellent comprehensive properties. Furthermore, the interaction between CS and pectin was closely related to the degree of methyl-esterification (DM), molecular weight (*M*_w_), and zeta potential of pectin. The low DM, *M*_w_, and high zeta potential of the low methyl-esterified pectin (LM) resulted in a denser internal structure of the bilayer film, stronger UV shielding performance, and stronger gas barrier ability. The high DM and *M*_w_ of the high methyl-esterified pectin (HM) endow the bilayer film with stronger mechanical properties, thermal stability, and antifogging property. The microstructural and spectroscopic analysis showed that there are hydrogen bonds and electrostatic interactions between the layers. Overall, the developed CS-pectin polyelectrolyte bilayer films provided potential applications for food bioactive packaging.

## 1. Introduction

As an important part of the food industry supply chain, food packaging acts as a protective layer or barrier layer for food, avoiding the impact of external adverse factors, such as spoilage microorganisms, chemical contaminants, oxygen, moisture, light, external pressure, etc. Packaging materials keep food safe from potential damage and degradation during storage and transport to extend its shelf life [1]. In the market, packaging materials are gradually enabling advanced packaging innovations such as active and smart packaging that help to regulate and monitor the quality of food during its shelf life [2]. Traditionally, many commonly used packaging materials are plastics derived from petroleum, which are widely used due to their excellent deformation properties and lighter weight compared to other materials, such as polyethylene and polypropylene [3]. According to statistics, global bioplastic production in 2020 was estimated to be 350 million tons, around 40% of which was consumed by the packaging industry [4]. Rising social awareness of ecological issues and strict regulations by regulatory agencies have created a need for the food packaging market to find alternatives to petroleum packaging materials. Naturally, biomass polymers have been considered as a solution to minimize the environmental impact of microplastics. Common natural polymer materials are mainly polysaccharides, proteins, lipids, and their combinations. Polysaccharide polymers show the advantages of a wide range of sources, low cost of raw materials, and stable properties [5].

Citrus pectin is a natural anionic polysaccharide in natural citrus tissue’s cell walls and is widely used as a food additive [6]. The properties of pectin vary with the degree of methyl-esterification (DM) and molecular weight (*M*_w_) and are often classified as high methyl-esterified (HM) and low methyl-esterified (LM) pectin. As a source of food packaging materials, pectin polymers exhibit low toxicity, good water solubility, gelling properties, and film-forming properties [7]. As natural anionic polysaccharides, both HM and LM pectin are negatively charged based on the polyelectrolyte properties of pectin. However, due to their disadvantages of poor moisture barrier, thermal, and mechanical properties, their practical application value as food packaging is relatively low [8]. For these problems, various research has come up with some solutions, such as blending with other polymers [7] and adding nanoparticles [9].

Layer-by-layer (LBL) casting technology is a method for the preparation of polyelectrolyte multilayer films based on the interaction between molecules with different charges, including electrostatic interactions, hydrogen bonding interactions, and hydrophobic interactions [10]. Intermolecular electrostatic and hydrogen bonding interactions were formed during polyelectrolyte assembly [11]. During the polyelectrolyte assembly, citrus pectin could participate in the supply of negative charges, while another polymer is required to provide positive charges to form self-assembly. Chitosan (CS) is a common cationic polysaccharide obtained from the extraction of chitin from shellfish processing waste. It presents good film-forming properties, satisfactory biocompatibility, and broad-spectrum antibacterial ability, but shows poor UV barrier properties, water vapor barrier properties, and mechanical properties, limiting its application in natural packaging materials [12].

CS and pectin are hydrophilic natural polymers, resulting in the formation of films that are highly hydrophilic and easily ruptured. The commonly used blending method was to mix film-forming solutions (FFSs) with good compatibility, in order to complement the properties through polymer characteristics [13]. Previous studies have shown that the physicochemical properties of thin films were closely related to the intermolecular interactions that existed between the thin film matrix [14]. However, if CS and pectin FFS were directly mixed, it would form gels or white flocculent precipitates. This largely affects the film formation process. As a result, it is impossible to remove the bubbles and achieve a dispersed state in the FFSs, resulting in non-uniform and poor-performance films [15]. It is assumed that the positively charged CS polyelectrolyte and the negatively charged pectin polyelectrolyte can form a polyelectrolyte bilayer film with better performance by the LBL casting method. The electrostatic interaction between the positively charged CS and the negatively charged pectin can enhance the physicochemical properties of the films [16]. The use of polyelectrolyte assembly to develop high-performance bilayer films deserves further exploration, in order to avoid gelation or precipitation caused by blending technology and prepare uniform and flat films. Polyelectrolyte bilayer films prepared by polyelectrolyte assembly have potential applications in the field of food packaging. Compared with the limited performance of monolayer films, bilayer films could better utilize the unique properties of each layer of the matrix to meet the requirements of multifunctional packaging materials [17]. According to previous reports, a unidirectional water-blocking film with a moisture-proof outer layer and a hydration inner layer can be formed by the LBL casting method [18]. With the support of polyelectrolyte assembly, the CS-pectin bilayer film is expected to avoid the generation of insoluble polymers when mixing the FFSs, while improving the properties of pure CS and pectin films. Moreover, the physicochemical properties, such as charge size, hydrogen bond-donor acceptor, DM, and *M*_w_, could be affected by the pectin kinds (HM and LM). Thus, the polyelectrolyte bilayer film formed by pectin and CS could show different performances. However, few studies have investigated the preparation of polyelectrolyte bilayers by different types of pectin as negatively charged polymers.

Thus, in this study, polyelectrolyte bilayer films of CS and four types of edible citrus pectin combinations were prepared by polyelectrolyte assembly and compared with CS and pectin monolayer films. The physicochemical properties of these monolayer and bilayer films, including film thickness, microstructural properties, optical properties, mechanical properties, barrier properties, and antifogging properties were characterized. Additionally, the assembly mechanism of polyelectrolyte bilayers of CS and different citrus pectin were explored. These results would provide an economical and convenient packaging combination of polyelectrolyte bilayers for potential food active packaging.

## 2. Materials and Methods

### 2.1. Materials

CS (deacetylation degree: 90%) was obtained from Jinan Haidebei Biotechnology Co., Ltd. (Jinan, China). Four commonly commercially used edible citrus pectin, including two types of LM pectin (L200, L201Y) and two types of HM pectin (H183, H182), were purchased from Yantai Andre Pectin Co., Ltd. (Yantai, China). The DE and degree of amidation (DA) of these four types of pectin were shown in Table 1. Acetic acid and glycerol were supplied by Sinopharm Chemical Reagent Co., Ltd. (Shanghai, China). Glycerol was purchased from China Medicine (Group) Shanghai Chemical Reagent Co. (Shanghai, China). PE (polyethylene) film was purchased from the local supermarket (Changsha, China).

### 2.2. M_w_ and Zeta Potentials of CS and Pectin

The relative molecular masses of CS and pectin were determined by the HPSEC method according to a previous method with slight modifications [19]. In detail, CS and pectin samples were prepared at a concentration of 1.0 mg/mL. HPSEC analysis was performed on an LC20 HPLC system (Shimadzu, Kyoto, Japan) with a TSKgel GMPWXL hydrogel column (Tosoh, Japan). Twenty microliters of the sample solution (10 mg/mL) were injected and eluted with 0.2 M NaCl at a flow rate of 0.6 mL/min. The columns and detector were maintained at 40 °C. The zeta potentials of CS and pectin were measured with a zeta potential analyzer (NanoBrook Omni from Brookhaven Instruments, New York, NY, USA) at a diluted concentration (1%, *w*/*v*).

### 2.3. Preparation of Films

The CS solution (2%, *w*/*v*) was obtained by mixing 2 g CS in 2% (*v*/*v*) acetic acid solution and kept at 25 °C for 12 h with stirring. Each kind of citrus pectin solution (2%, *w*/*v*) was prepared by dissolving 2 g of corresponding pectin powder in distilled water with stirring at 25 °C for 12 h. Afterward, glycerol (25%wt of the CS or pectin) was also added to the solution as a plasticizer to improve the flexibility and workability of the films. The film-forming solutions were sonicated for 30 min before used to remove bubbles. The CS and pectin films were prepared by the solution casting method. The CS FFS and pectin FFSs were cast onto a Plexiglas plate (10 cm × 10 cm) and allowed to dry in a drying oven at 45 °C, respectively. The CS-pectin films were prepared according to the method of Ferreira et al. [17] with some modifications. First, each kind of pectin solution was poured onto a Plexiglas plate (10 cm × 10 cm) and allowed to dry in a drying oven at 45 °C until a firm yet adhesive surface was obtained. The CS solution was then spread on top of the pectin layer, and the bilayer was dried in a drying oven at 45 °C for 14 h and subsequently removed from the Plexiglas plate. The obtained films were stored in a desiccator at 50% relative humidity and 25 °C for at least 48 h.

### 2.4. Characterization of Films

#### 2.4.1. Measurement of Thickness and Mechanical Properties

Film thickness was measured using a digital micrometer (Sanliang Measuring Tools Co., Ltd., Dongguan, China) with an accuracy of 0.001 mm. The thickness was measured at 6 randomly selected locations of each sample and the mean values were determined.

The tensile strength (TS) and the elongation at break (EAB) of film samples (80 mm × 10 mm) were measured using an electronic tensile testing machine (Labthink Co., Ltd., Jinan, China). Samples were measured in the tensile mode with a separation of 50 mm and a stretching rate of 10.0 mm/min [20].

#### 2.4.2. Color

The color values were evaluated using the Hunter LabScan colorimeter (MS/S-4500L, Hunter Associate Laboratory Inc., Reston, VA, USA). Before the measurements, calibration was performed with a standard white plate (L* = 93.48, a* =−0.65, b* = 1.91). The color values of *L* (lightness), *a* (red/green), and *b* (yellow/blue) were applied with the calculation of total color differences (Δ*E*) and whiteness index (WI), with the following Equations (1) and (2) [21]:

(1) $$\Delta E=\sqrt{{\left(L*\;-\;L\right)}^{2}+{\left(a*\;-\;a\right)}^{2}+{\left(b*\;-\;b\right)}^{2}}\;$$

(2) $$\mathrm{WI}=100\;-\sqrt{{\left(100\;-\;L\right)}^{2}+a^{2}+b^{2}}$$

#### 2.4.3. Optical Properties

The film was attached to the inside of the cuvette and the transmission spectra of the films were determined using a UV-visible spectrophotometer (UV-1800, Shimadzu, Kyoto, Japan) from 200 to 800 nm. The opacity of the film was measured at 600 nm and calculated by Equation (3) as follows [22]:

(3) $$\mathrm{Opacity}=-{log(T}_{600})/d$$

where the *T*_600_ is the transmittance at 600 nm and *d* is the average thickness of the film (mm).

#### 2.4.4. Microstructure

The surface and cross-section morphological features of films were obtained by scanning electron microscopy (SEM, Carl Zeiss, Germany) at 10 kV accelerating voltage. In detail, the films were fractured in liquid nitrogen and were coated with chromium sputtering to enable the observation of surface and cross-section. The microcosmic morphology was obtained at 1000× magnifications to enable the observation of the surface and cross-section.

The surface roughness of films was obtained by atomic force microscopy (AFM, Bruker, Germany). The surface average roughness (*R*_a_) and root-mean-square roughness (*R*_q_) of films were calculated by Nanoscope analysis software (version 1.8).

#### 2.4.5. Moisture Content (MC) and Water Solubility (WS)

The MC and WS of the film were determined based on the method with some modifications [18]. The film samples were weighed (*M*_1_) and dried to a constant weight (*M*_2_) in an oven maintained at 105 °C. The MC of the films was calculated with the following Equation (4):

(4) $$\mathrm{WC}\;=\;[\left(M_{1}\;-\;M_{2}\right)\;/\;M_{1}]\;\times \;100\%$$

The film samples were immersed in 50 mL of deionized water at room temperature for 24 h and dried at 105 °C to a constant weight (*M*_3_). The WS was calculated with the following Equation (5):

(5) $$\mathrm{MS}\;=\;[\left(M_{2}\;-\;M_{3}\right)\;/\;M_{2}]\;\times \;100\%$$

#### 2.4.6. Water Vapor Permeability (WVP)

The WVP of the film was determined based on the method with some modifications [20]. Film samples were sealed in a test vessel (2.8 cm in diameter, 11.5 cm in height) containing 12 g anhydrous calcium chloride. The test vessel containing the film sample was weighed initially and then placed in an environment with a temperature of 25 °C and relative humidity of 75% for 48 h. The WVP of films was calculated with the following Equation (6):

(6) WVP=(ΔM × d) / (A × t × ΔP)

where Δ*M* is the increased weight of the test vessel (kg), *d* is the thickness of films (m), *A* is film effective area (m^2^), *t* is the equilibrium time (s), Δ*P* is the penetrating area of difference of water vapor pressure (Pa).

#### 2.4.7. Oxygen Permeability (OP) and Carbon Dioxide Permeability (CDP)

The OP and the CDP of the film were determined based on the method reported by Kim et al. [23]. The OP was evaluated by the method of deoxidizer absorption. In detail, film samples were sealed on the top of the test vessel (2.8 cm in diameter, 11.5 cm in height) containing 5 g deoxidizer. The CDP was evaluated in accordance with the method of alkali absorption. Film samples were sealed on the top of the test vessel containing 5 g potassium hydroxide. The test vessel containing the film sample was weighed initially and then placed in an environment with a constant temperature of 25 °C and relative humidity of 75% for 48 h. The OP and CDP of films were calculated as follows:

(7) OP =(ΔM × d) / (A × t × ΔP)

(8) CDP=(ΔM × d) / (A × t × ΔP)

where Δ*M* is the increased weight of the test vessel (kg), *d* is the thickness of films (m), *A* is film effective area (m^2^), and *t* is the equilibrium time (s).

#### 2.4.8. Thermogravimetric Analysis (TGA)

The TGA test was carried out using a thermogravimetric analyzer (Netzsch, Germany). The samples were heated from 25 to 600 °C under a nitrogen atmosphere. The heating rate was 10 °C/min. The TGA and derivate thermogravimetric (DTG) analyses were performed on the different films.

#### 2.4.9. X-ray Diffraction (XRD)

The XRD of the film sample was analyzed using an X-ray diffractometer (Bruker D8 Advance, Germany) under 40 kV and 30 mA in the 2θ range of 5° to 45°.

#### 2.4.10. Fourier Transform Infrared (FT-IR) Spectroscopy

The FT-IR spectra of film samples were determined using a spectrometer (Nicolet, Nexus 410, Woodland, CA, USA). The FTIR spectral analysis was performed from 4000 to 400 cm^–1^ with a resolution of 4 cm^−1^.

#### 2.4.11. Antifogging Properties

Thermal antifogging test: a beaker filled with 50 °C water (about 60 mL) was covered with commercially available PE film, pectin monolayer films, CS monolayer film, and LBL-CS-pectin bilayer films, and it was maintained in a water bath to evaluate thermal antifogging performance. The fogging of the film surface was observed and recorded after 30, 60, 90, 120, and 240 min, respectively.

Cold antifogging test: a beaker was covered with commercially available PE film, pectin monolayer films, CS monolayer film, and LBL-CS-pectin bilayer films, and it was placed in a –20 °C refrigerator for 2 h. The fogging of the film surface was observed and recorded after the film was exposed to room temperature for 30 s.

### 2.5. Statistical Analysis

All experiments were conducted in three replicates. Statistical analyses were performed using the SPSS 20.0 software (Chicago, IL, USA). All the data were analyzed by one-way analysis of variance (ANOVA) with Duncan’s test and expressed as the mean ± standard deviation. Differences were considered to be statistically significant with *p* < 0.05.

## 3. Results and Discussion

### 3.1. M_w_ Distribution and Zeta Potential of CS and Pectin Film-Forming Solutions

**Figure 1:**
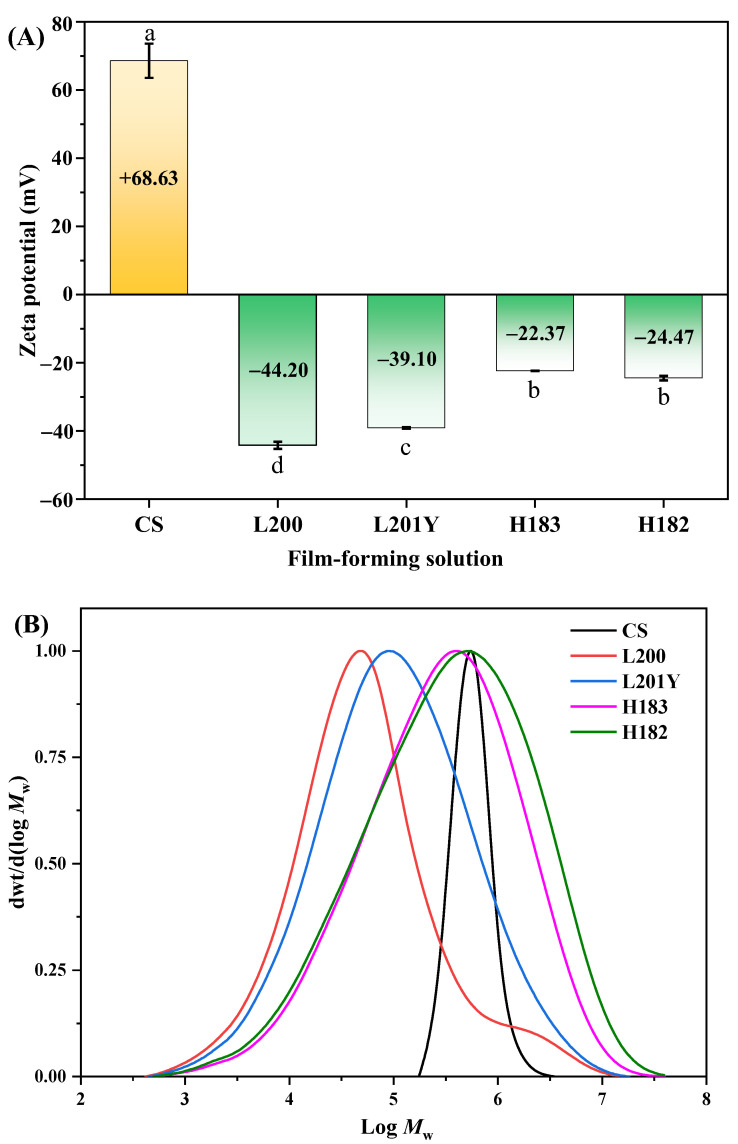
Zeta potential (**A**) and *M*_w_ distribution (**B**) of CS and pectin film-forming solutions. Different letters within the histogram indicate significant differences (*p* < 0.05) in value by using Duncan’s test.

The zeta potential values of CS and pectin FFSs were shown in Figure 1A. The zeta potentials of CS and pectin samples were as follow: CS (+66.63 mV), L200 (–44.20 mV), L201Y (–39.10 mV), H183 (–22.37 mV), and H182 (–24.47 mV), respectively. Since CS was dissolved in the acetic acid solution, its amino group (–NH_2_) was ionized into –NH_3_^+^, resulting in a positive zeta potential value. The carboxyl groups (–COOH) in pectin are ionized to –COO^−^, resulting in a negative zeta potential value. Lower DM resulted in more –COOH in LM pectin compared to HM pectin. The analysis of zeta potential showed that the absolute value of zeta potential decreased with the increase of DM values of pectin. Therefore, the electrostatic attraction could be formed between CS and pectin and its intensity could be affected by the DM values of pectin [9].

Figure 1B showed the *M*_w_ distribution of CS and different types of pectin. The distribution of molecular weight peaks was in the order of magnitude of 10^3^–10^7^. Both CS and the four types of pectin showed a unimodal distribution with mono-dispersity. The physicochemical properties of pectin mainly depended on DM, *M*_w_, and the degree of structural branching. The *M*_w_ and *M*_n_ values of the CS and pectin samples were as follows (Table 1): CS (619.50 and 514.00 kDa), L200 (87.46 and 16.41 kDa), L201Y (369.84 and 27.12 kDa), H183 (873.34 and 54.07 kDa), and H182 (1243.64 and 53.34 kDa), respectively. The *M*_w_ of pectin samples was in the order of H182 > H183 > L201Y > L200, which was in the range of the previous result of citrus pectin [24]. The polydispersities of the pectin samples were as below: L200 (5.33), L201Y (13.64), H183 (16.15), and H182 (23.31), respectively. This indicated that the different pectin types showed diversified *M*_w_ distributions, and the high polydispersity could be due to its extensive branched structure [25].

### 3.2. Color and Opacity

The color and opacity of packaging materials are important attributes to evaluate the visual appearance and consumer acceptance of the product. CS and CS-pectin with different DM and *M*_w_ were composited into films, and the effects on the optical properties of the films were shown in Table 2. In terms of overall physical appearance, the film was generally smooth, uniform, and transparent. Compared with pectin films and bilayer films, the CS film exhibited the highest *L* and WI values, while showing the lowest Δ*E* value (*p* < 0.05), indicating that the CS film was more transparent and brighter. For *a* and *b* values, the difference between the 4 pectin films was not significant (*p* > 0.05), nor was there a significant difference between the polyelectrolyte bilayer films composed of pectin (*p* > 0.05), respectively. This suggested that differences in DM and *M*_w_ of pectin properties do not affect the appearance and color properties of pectin films. Furthermore, there was no significant difference between the CS film, the pectin films, and the CS-pectin bilayer films (*p* > 0.05), which indicated that all the films showed the same color and were colorless and transparent [12].

The opacity of films is an inverse measure of transparency. A higher opacity indicates a worse visual characteristic of the product obtained by the consumers [26]. The opacity of the different films was shown in Table 2. The transparency of the CS film measured at 600 nm was 1.19 mm^−1^, while that of the pectin films varied from 1.12 mm^−1^ to 1.16 mm^−1^. For pectin films, there were no significant differences in opacity values among the four types of pectin with different DM and *M*_w_ (*p* > 0.05). It could be observed that the transparency of the four types of pectin films was higher than that of the CS films, and the pectin polymer macromolecules have higher solubility and dispersibility in FFSs because the *M*_w_ of the four types of pectin is lower than that of CS [27]. The opacity value of the CS-pectin bilayer films was in the range of 0.97–1.03 mm^–1^. There was no significant difference in the transparency between the polyelectrolyte bilayer films formed with different types of pectin (*p* > 0.05).

### 3.3. Light Barrier Properties

**Figure 2:**
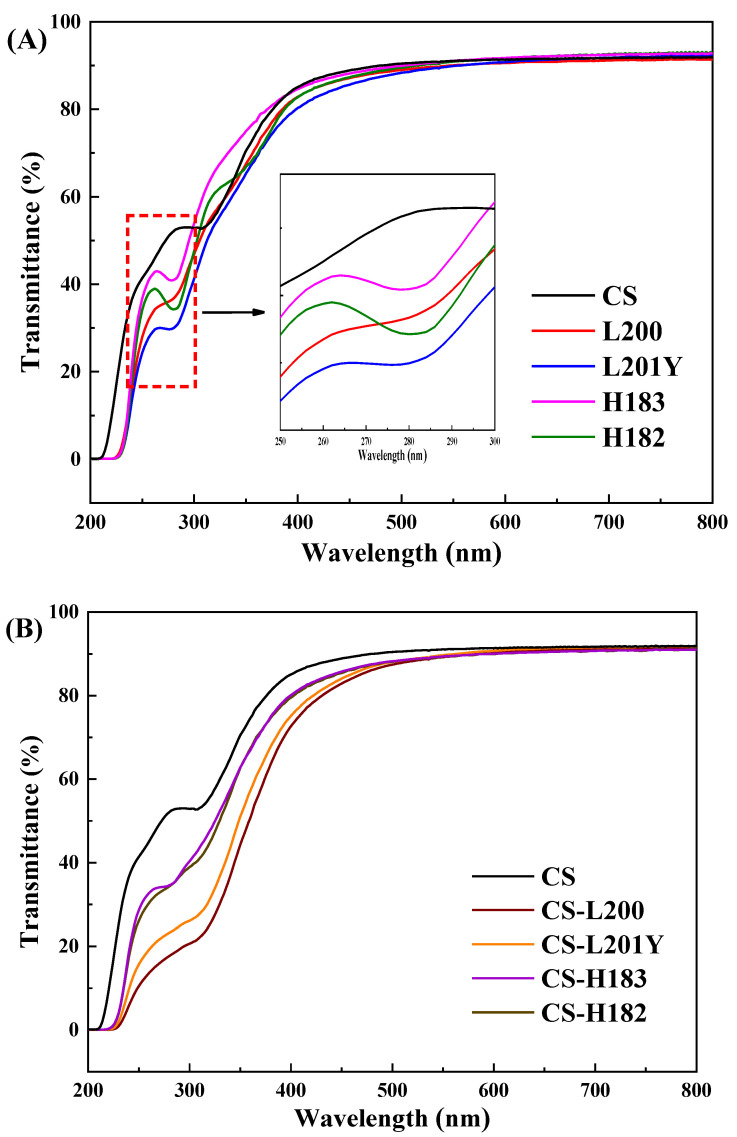
UV-vis transmittance of different monolayer (**A**) and bilayer (**B**) films. The subfigure of (**A**) was enlarged interpretation of the intersection regions of UV-vis transmittance curves from 250 to 300 nm.

For food packaging materials, the shielding effect of ultraviolet light is an important film property. The UV transmittance of the prepared films were shown in Figure 2. The CS film presented poor UV blocking ability due to the lack of UV-Vis absorbing groups in its structure [28]. The pectin films showed absorption peaks in the ultraviolet range (below 300 nm) without specific absorption in the visible range. The light in the ultraviolet range could be absorbed by UV-absorbing groups such as the amide groups of the LM pectin [29]. The UV transmittance of the LM pectin film at 260 nm was 33.46% for the L200 film and 29.08% for the L201Y film, which was lower than those of the HM pectin film, with the UV transmittance of 42.27% for the H183 film and 38.76% for the H182 film, respectively. The UV shielding function of the pectin bilayer film was further enhanced after polyelectrolyte assembly, with 86.42% for the CS-L200 film and 80.83% for the CS-L201Y film UV light-shielded in the CS-LM film. It indicated that the UV shielding effect was significantly higher than that of the CS-HM bilayer film (*p* < 0.05). This can be explained by the enhanced intermolecular interactions of polyelectrolyte assembly. Low DM resulted in stronger intermolecular hydrogen bond interaction between –OH and –COOH in LM pectin and –NH_2_ and –OH in CS. Likewise, the higher zeta potential of LM pectin endows more film surface charges, resulting in strong electrostatic interaction [18]. Hydrogen-bonding interactions and electrostatic interactions facilitated the formation of dense film structures during the polyelectrolyte assembly of LM pectin and CS [10]. The dense structure of the bilayer film was conducive to improving the UV shielding function of the film [30]. Therefore, L200 of LM pectin showed stronger UV blocking ability in assembly, which was related to higher zeta potential and *M*_w_ than L201Y. A previous study also reported that CS interacted with anionic polysaccharides through polyelectrolyte assembly, which reduced the UV transmittance of the film and effectively protected food from UV damage [26].

### 3.4. Film Morphology

**Figure 3:**
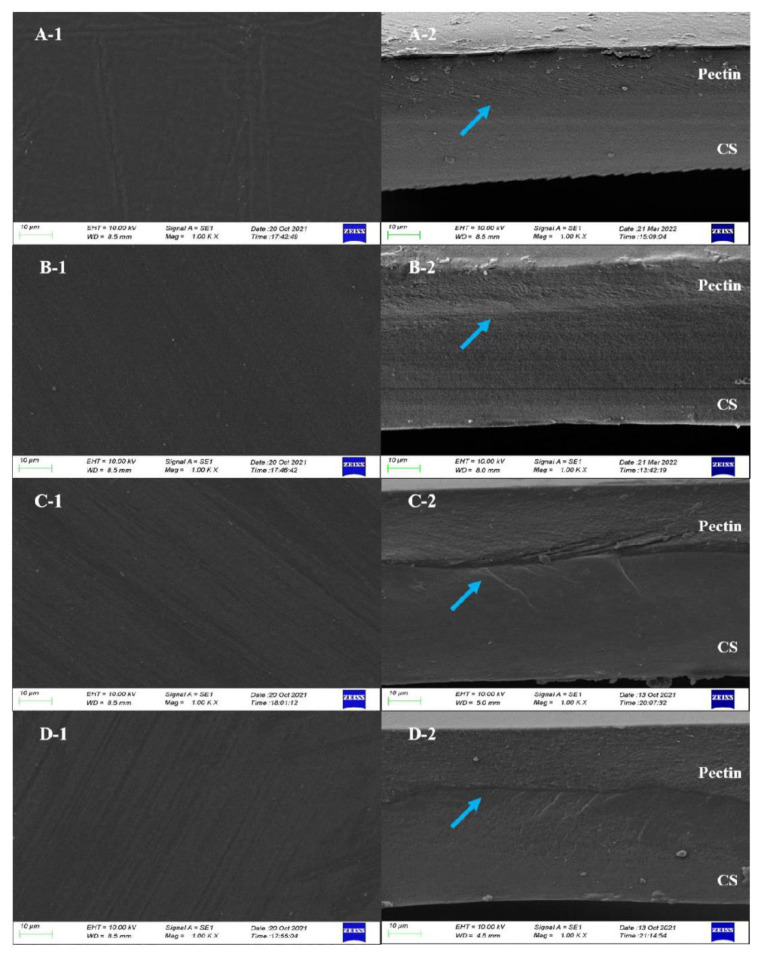
SEM micrographs of surface (1) and cross-section (2) of CS-pectin bilayer films ((**A1**,**A2**): CS-L200, (**B1**,**B2**): CS-L201Y, (**C1**,**C2**): CS-H183, (**D1**,**D2**): CS-H182). The boundary of bilayer was pointed by the blue arrow.

Microstructural morphologies of the films were characterized using SEM and AFM. The SEM images for the surface and cross-sections of the CS-pectin films were displayed in Figure 3. It can be observed that the surface roughness of different pectin was not uniform, indicating less homogeneity of the components in the films due to different DM and *M*_w_ (Figure 3A). Additionally, no pores or cracks were observed on the surfaces of the four polyelectrolyte bilayer films (Figure 3A). Both the LM pectin side and the HM pectin side of the polyelectrolyte bilayer film exhibited homogeneity, despite the presence of granular crystalline material in both film matrices. In sharp contrast, previous findings showed that the LM pectin was higher than the HM pectin in terms of densification of the constituent materials, due to the more polar groups contained in the LM pectin, which resulted in higher densities in the film fraction [31]. As shown in Figure 3B, the SEM image of the cross-section of the polyelectrolyte bilayer film showed a dense, continuous, and fracture-free structure. In the cross-section of the bilayer film, the CS layer exhibited a larger thickness compared to the pectin layer. The thickness of the composite film depends on the nature and composition of each component. According to Dai et al. [32], CS had strong water retention and swelling ability. The presence of numerous hydrophilic groups and water storage sites in CS films could result in high swelling of the film-forming matrix [33]. While the pectin film matrix also had strong hydrophilic properties, the matrix volume did not expand by water absorption [34]. The determination of moisture content (Table 3) further supported our above arguments. Therefore, when dried in a constant volume container, the CS matrix with a high volume might produce a thicker layer than the pectin matrix. The pectin layer in the bilayer film exhibited a dense porous structure with uniform pores of similar size distributed in the film matrix, which could be attributed to the highly uniform dispersion of pectin molecules in FFS. Previous studies had shown that the porous composite structure was mainly derived from the strong gas-holding ability brought about by the interfacial activity of FFS, and the polymer could not form a continuous structure during the drying process to form pores [35]. 

In comparison to the pectin, it was found that the rough structure of the cross-section of the pectin layer was not directly related to the *M*_w_ of the pectin particles. The low *M*_w_ of the L200 pectin component and the highest *M*_w_ of the H182 pectin component showed similar roughness. The cross-sectional morphologies in Figure 3B showed that the CS-LM (Figure 3(A-2,B-2)) and CS-HM (Figure 3(C-2,D-2)) films were somehow different. The CS layer in the film was tightly combined with the LM pectin layer, blurring the boundary and forming a dense and uniform bilayer structure between the two substrates. This confirmed that two-layer substrates with positive and negative opposite charges can achieve high compatibility between the two layers through polyelectrolyte assembly [10]. However, there was a demarcation phenomenon in the CS-HM films. The CS-HM films showed distinct boundary layers, such as longitudinal and transverse grains, leading to a loose bilayer interfacial structure of the films. This phenomenon could be attributed to the difference in the strength of molecular interactions involved in the assembly of the CS with pectin with different DM. As shown in Table 1, since the DM of HM pectin was higher than that of LM pectin, the amounts of polar groups and hydrogen bonds in HM pectin were less than that of LM pectin, and the hydrogen-bonding molecular interaction existing between the HM pectin layer and CS layer was also weaker [31]. Interestingly, the zeta potential value of the pectin matrix represented the difference in electronegativity in Figure 1. The zeta potential value of the LM pectin film was −44.2 mV for the L200 film and −39.1 mV for the L201Y film, which was higher than those of the HM pectin film, with the zeta potential value of −22.37 mV for the H183 film and −24.47 mV for the H182 film, respectively. The zeta potential of the LM pectin was higher than that of the HM pectin, resulting in a more robust electrostatic interaction with the polycationic matrix CS for polyelectrolyte assembly. As the electronegativity increased, the tendency to accept electrons also increased, which resulted in a tighter polyelectrolyte assembly structure combined with the CS layer [36,37]. This explained the compatibility issue between the CS and the demarcation between the pectin layers of H183 and H182. The SEM images indicated that the differences in the DM and zeta potential of the pectin components could affect the hydrogen bonding and electrostatic interactions with the CS components. The low DM and high zeta potential of pectin could lead to a denser and more compatible bilayer film structure.

**Figure 4:**
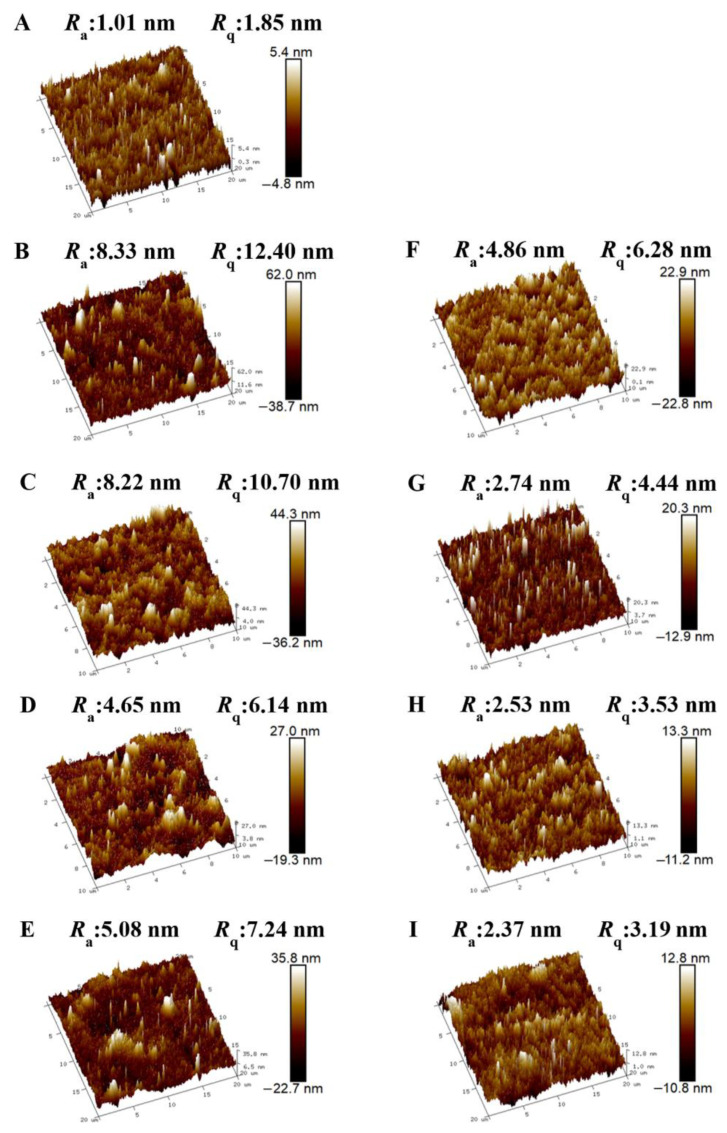
AFM 3D images, average roughness (*R*_a_), and root-mean-square roughness (*R*_q_) of different monolayer and bilayer films ((**A**): CS, (**B**): L200, (**C**): L201Y, (**D**): H183, (**E**): H182, (**F**): CS-L200, (**G**): CS-L201Y, (**H**): CS-H183, (**I**): CS-H182).

The three-dimensional surface topography images and the values of *R*_a_ and *R*_q_ obtained by AFM are commonly used to characterize roughness. As shown in Figure 4, the CS film exhibited a smooth and dense surface structure, and the values of *R*_a_ and *R*_q_ were 1.01 and 1.85 nm, respectively. In contrast, the surfaces of the pectin films were rougher than that of the CS film. The film surface morphology depends on the composition of the substrate, the polymer structure of the substrate, the concentration, and the dispersion of the FFSs. In the process of preparing the FFSs, it could be observed that the pectin FFSs showed poor dispersibility with slight aggregation appearing in the aqueous solution [38]. Interestingly, the surface roughness of the LM pectin films was higher than that of the HM pectin films. LM pectin was obtained by demethoxylation of HM pectin with a complex branched structure. The DM of LM pectin was lower than that of HM pectin, and its folded molecular structure contained more –COOH groups, resulting in stronger intramolecular hydrogen bonding. Therefore, the solubility of LM pectin was lower than that of HM pectin, resulting in a rougher internal structure of the film [39,40]. However, there was no obvious difference between the surface roughness of the L200 and L201Y in LM pectin, and there was also no obvious difference between the H183 and 182 in HM pectin. As shown in Figure 4F–I, the values of *R*_a_ in bilayer films were in the range of 2.53–4.86 nm, and the values of *R*_q_ were in the range of 3.53–6.28 nm, respectively. After polyelectrolyte assembly, the deposition of pectin components appeared to improve the surface uniformity of films. Compared with the corresponding pectin monolayer film, the roughness *R*_a_ of the CS-pectin bilayer film was reduced by 41.66%, 66.67%, 45.59%, and 53.35%, and the roughness *R*_q_ was reduced by 49.35%, 58.50%, 42.51%, and 55.94%, respectively. This confirmed the deposition of pectin components on CS components during polyelectrolyte assembly, and the pectin-side surface of bilayers exhibited few protrusions and inhomogeneities. It had been previously reported that pectin was usually present in polysaccharide films in the form of polymers, linear chains, and branched chains [41]. As shown in the AFM images, a large number of polymers and linear chains were observed in the pectin films. The polymer and linear chains on the surface of the adhesive film after polyelectrolyte assembly were more finely dispersed, which indicated that the combination of polyelectrolyte assembly can obtain films with good surface uniformity. During the assembly of the bilayers, the decreasing rate of roughness and pectin with different DM was not significantly associated with CS assembly. Although the higher potential of the pectin particles in the system led to stronger intermolecular stronger electrostatic interaction forces, which affected the roughness of the pectin side, the roughness of the pectin surface might be more affected by dispersibility and solubility [40,42].

### 3.5. Moisture Content, Water Solubility, and Barrier Properties

The sensitivity of biopolymer packaging materials to water has important implications for the shelf life of packaged foods. The moisture content (MC) and water solubility (WS) of films were shown in Table 3. MC reflects the total free volume of water molecules in the film structure network. CS films showed the highest MC, which was related to the hydrophilicity of CS. The presence of hydroxyl and amino groups in CS increased the availability of hydrophilic groups, improving the affinity of CS films toward water molecules [43]. WS indicates the integrity of the film under aqueous conditions. Both citrus pectin and CS are hydrophilic polysaccharide materials. The WS of the four citrus pectin films was in the range of 60–80%. According to Çavdaroğlu et al. [42], the films composed of crude fig pectin showed almost 71–72% solubility, while the films of commercial citrus and apple showed 100% solubility. That might be related to the purity and heterogeneity of pectin. The differences in DM and *M*_w_ of pectin materials posed no significant effect on the solubility of pectin films (*p* > 0.05). The solubility of CS-pectin bilayer films in water was significantly reduced compared with pectin monolayer films (*p* < 0.05). This resulted in improved integrity and water resistance of the polyelectrolyte films under aqueous conditions.

WVP of packaging materials was usually used to evaluate the ability of packaged food to exchange moisture with the outside world in a high-humidity environment [44]. The WVP values of the CS, pectin, and CS-pectin films was shown in Table 3. The WVP values of the LM pectin films, HM pectin films, and polyelectrolyte bilayer films were significantly lower than that of CS film, respectively (*p* < 0.05). For the pectin films, increasing DM posed no significant effect on WVP values (*p* > 0.05). The CS film with the highest WVP was 1.46 × 10^–10^ g·m^−1^·s^−1^·Pa^−1^. This result could be attributed to the fact that the polysaccharide chains in CS contain polar groups such as amino, hydroxyl, and carboxyl groups. Polar groups promoted the interaction between polysaccharide chains and water molecules, resulting in the adsorption and desorption of water molecules on the films [45]. As shown in Table 3, the WVP values of the CS-pectin films were higher than that of the pectin monolayer films but lower than that of the CS monolayer film. The WVP values of the CS-LM films were lower than that of the CS-HM films (*p* > 0.05). This result could be related to the DM and zeta potentials of pectin. Compared to HM pectin, LM pectin contained fewer –OCH_3_ and thus had –COOH to interact with –NH_2_ in CS [17]. This phenomenon led to stronger adhesion between the LM pectin layer and CS layer during polyelectrolyte assembly, and the hydrogen bonding interaction force between the layers reduce the exposure of hydrophilic groups, lowering the water vapor adsorbed on the film surface and weakening the hydrophilicity of the film [46]. Likewise, pectin molecules folded easily to form intramolecular hydrogen bonds. With the increase of DM, the hydrogen bonding in the pectin molecule weakened, and the HM pectin showed higher hydrophilicity [47]. This deduction could also be confirmed by the results of zeta potential as shown in Figure 1. With high zeta potential, L200 and L201Y pectin endowed the surface of film components with a large number of negative charges, thus L200 and L201Y pectin assembled with CS through strong electrostatic interactions during polyelectrolyte assembly. Under the action of a strong Coulomb force, the structure of the bilayer film could become denser, providing a more tortuous water vapor diffusion path [45]. The results of the WVP of the bilayer films were consistent with the SEM results of this study. As shown in the cross-section of the CS-pectin bilayer film, the two layers of the CS-LM film were tightly bound without obvious cracks and component separation, while the cracks at the interface of the two layers could be observed in the CS-HM film (Figure 3). This suggested that the compatibility between film substrates directly affects the water vapor barrier capability of the film. The CS-L200 film and CS-L201Y film showed lower WVP values in food packaging applications, which could effectively reduce the water loss of the packaged food.

The oxygen and carbon dioxide barrier capacity in food packaging materials is an important indicator, which can participate in regulating the concentration of oxygen and carbon dioxide in the environment to achieve the purpose of preservation [28]. As shown in Table 3, the OP and CDP values of the bilayer film were lower than those of the monolayer film, indicating that the bilayer film has stronger oxygen and carbon dioxide barrier properties. This phenomenon could be explained by the fact that the polar interaction of the interfacial polymer during polyelectrolyte assembly led to the formation of a dense structure of the two-layer film matrix [48]. The polyelectrolyte assembly formed intermolecular bonding at the interlayer interface in the film, which effectively filled the voids in the polymer macromolecular network structure, and the more tortuous transport path hindered the flow of oxygen and carbon dioxide [49]. Among the bilayers composed of the four types of pectin, only the CS-L200 bilayer film was significantly lower than the CS and L200 monolayer films in terms of OP and CDP values (*p* < 0.05). The higher the number of negative charges in pectin, the stronger the electrostatic interaction with the positive charges of CS. The L200 with the highest zeta potential formed a highly dense bilayer film structure with CS under the action of Coulomb force. It was consistent with the results of SEM (Figure 3). There were no significant differences in OP and CDP values between bilayer films (*p* > 0.05), indicating that the DM, *M*_w_, and zeta potential of pectin posed no effect on the assembly of pectin and CS into films. From the perspective of gas transport differences, the ratio of CDP to OP of bilayer films was higher than that of monolayer films, and the ratios of the CS-H183 and CS-H182 films were significantly improved. According to Zhang et al. [28], unlike conventional plastic films, a higher CDP to OP ratio in the film resulted in a higher carbon dioxide gas exchange rate in the film, which was beneficial to inhibit the respiration of fruits and vegetables. Previous studies also had shown that appropriate values of OP and CDP can effectively suppress anaerobic and aerobic respiration rates and prolong food shelf life [50].

### 3.6. Thickness and Mechanical Properties

The thickness of monolayers and bilayers based on the CS, pectin, and CS-pectin was shown in Table 4. The thickness of films ranged from 0.062 to 0.082 mm. The thickness of the CS (0.082 mm) film was significantly higher than that of the pectin films (*p* < 0.05). In the pectin films, there was no significant difference between the thickness of LM pectin and HM pectin (*p* > 0.05), confirming that the DM and *M*_w_ of pectin do not affect the thickness of the films. After polyelectrolyte assembly, the thickness of the CS-pectin bilayer films also did not increase significantly compared with pectin monolayer films (*p* > 0.05), which were lower than the intermediate values of both polymer layers. The thickness of the bilayer film is related to the interaction between the film constituents [51].

Food packaging materials need to show certain mechanical properties in order to resist external pressure and maintain packaging integrity during food transportation and storage [52]. The TS and EAB of the films were shown in Table 4. The TS of the monolayer CS film was 49.15 MPa and the EAB was 4.70%, respectively. Interestingly, each of the TS and EAB of the pectin films was positively related to its *M*_w_. In contrast to this result, previous research results indicated that the LM pectin films presented stronger mechanical properties than the HM pectin films. This observation was attributed to the fact that the LM pectin film contains more polar groups, resulting in a higher number of hydrogen bonds and a tighter internal structure [53]. However, the results of this study could be explained by the results of *M*_w_ and previous studies. According to Yan et al. [54], the apparent viscosity of the pectin solution was positively correlated with *M*_w_ and DM of the pectin polymer. Previous studies indicated that pectin polymers with higher DM and *M*_w_ showed longer chain conformations. The longer the chain length, the stronger the intermolecular entanglement or interaction, which increases the viscosity [40]. Due to the higher viscosity of the FFSs, the polymer chains were entangled with each other to form the network structures by stronger intermolecular hydrogen bonds, which resulted in the greater mechanical properties of films [55]. As shown in Table 4, the mechanical properties of the films were greatly improved after polyelectrolyte assembly. The TS and EAB values of the CS-pectin bilayer films were significantly higher than those of the corresponding pectin monolayer films (*p* < 0.05), respectively, and the mechanical properties of the CS-HM bilayer films were higher than those of the CS monolayer films. After polyelectrolyte assembly, the TS value of the bilayer film was increased by 21.29% and the EAB value was increased by 10.37% compared with the CS monolayer film, respectively. This improvement in mechanical properties can be attributed to the opposite charges of CS and pectin, and the formation of hydrogen bonds between –NH_2_ and –OH of the CS layer and –OH and –COOH of the pectin layer, resulting in the interaction of Coulomb and ionic-dipole forces [27]. Meanwhile, polyelectrolyte assembly increases the contact area between CS and pectin components, which was beneficial to enhance the interaction and compatibility between films [56]. In the bilayers, LM pectin with higher zeta potential bound more tightly to CS, while the CS-HM bilayer films showed higher TS and EAB values due to higher *M*_w_ and DM of HM pectin. This result proved that the mechanical properties of the bilayer films were more affected by the physical properties of the film-forming components than by the interactions between the components. As shown in the SEM results of this study, the two-layer matrix of the bilayer films forms uniformly fitted structures with strong interfacial adhesion. During the stretching process of the instrument, no layer separation phenomenon was observed in the bilayer film. It showed that the assembly has strong stability, and the CS-HM bilayer film shows a good practical application effect as an anti-mechanical external force packaging material.

### 3.7. Thermal Analysis

**Figure 5:**
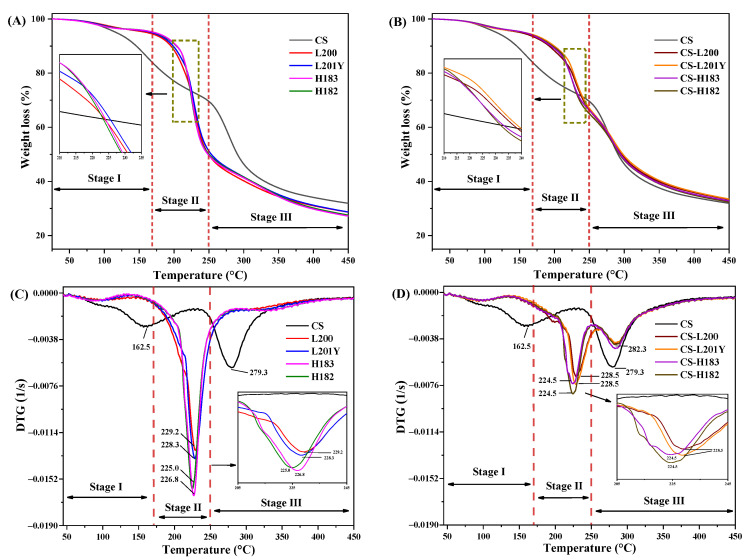
TGA curves and the relative derivative curves of different monolayer and bilayer films. The subfigures of A-D were enlarged interpretation of the intersection regions of the curves of stage II in thermal degradation.

During the preparation and application of packaging materials, the thermal stability of packaging films under high-temperature conditions was also an important indicator to consider [30]. Thermal decomposition properties of the CS, pectin monolayer films, and CS-pectin bilayer films were obtained by TGA and DTG. As shown in Figure 5A,B, the thermal decomposition curves of the films can be divided into three stages. In stage I (50 °C–170 °C), the reduction in mass was mainly attributed to the evaporation of free water and acetic acid from CS [16]. In stage II (170 °C–250 °C), weight loss was due to the degradation of glycerol and polymer pectin [40]. In stage III (250 °C–450 °C), the thermal decomposition of CS involved the depolymerization of sugar rings [48]. After high-temperature thermal decomposition, the average residual rate of the bilayer film (33.52%) was higher than that of the CS (31.92%) and pectin film (28.02%). The improved thermal stability was caused by the interaction between the polyelectrolyte components. As shown in Figure 5C,D, the DTG curve more clearly showed the decomposition rate of each stage in the thermal decomposition process. The results showed that the thermal decomposition rate of the bilayer film in stage II is significantly lower than that of the pectin monolayer film, while the maximum degradation temperature in stage III is higher than that of the CS monolayer film. This showed the fact that the thermal stability of the polymer film matrix can be improved after assembly [26]. Polyelectrolyte assembly promoted hydrogen bonding and electrostatic interactions between bilayer substrates, increasing film compaction through interactions and improving thermal stability [48]. In stage II, the maximum thermal decomposition rate of LM pectin was higher than that of HM pectin. The polyelectrolyte assembly narrowed the gap between the LM pectin film and the HM pectin film. It promoted the formation of hydrogen bonds between LM and CS, which made the molecular chain structure of LM compact and difficult to thermally decompose.

### 3.8. XRD

**Figure 6:**
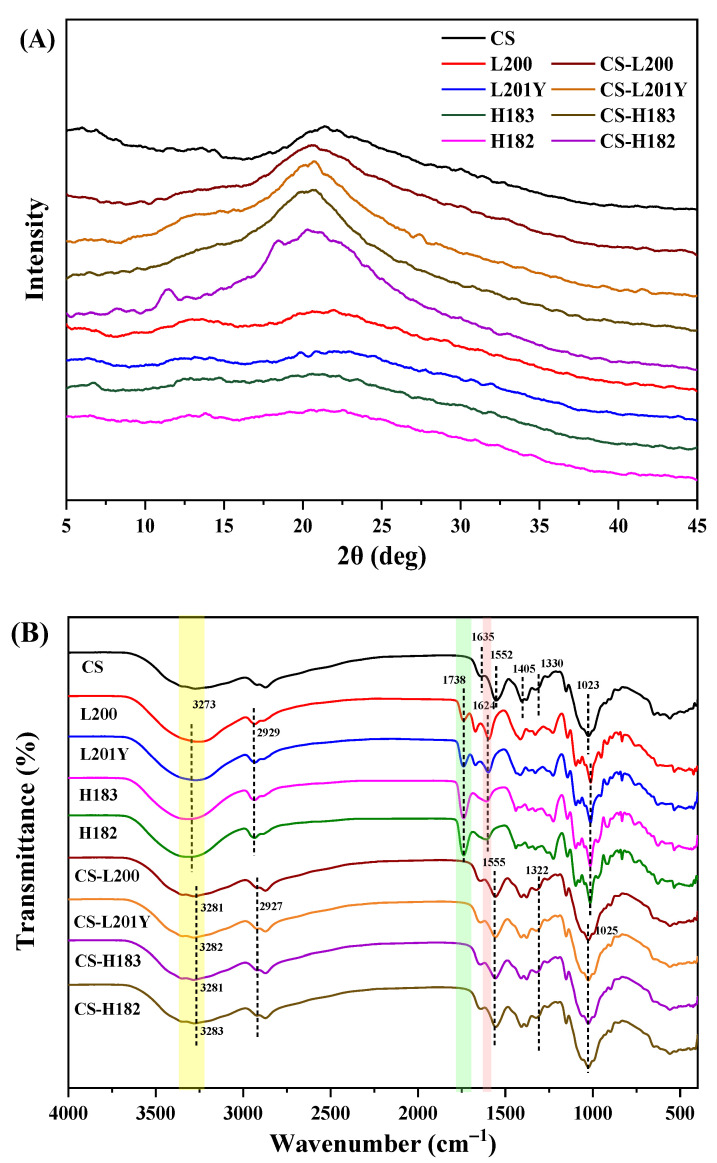
XRD (**A**) and FT-IR (**B**) spectra of different monolayer and bilayer films.

As shown in Figure 6A, no diffraction peaks were observed in the four pectin-based monolayers, while a characteristic diffraction broad peak appeared at 20.5° in the CS monolayer, which agreed well with the previous results reported by Neto et al. [57]. The films prepared by polyelectrolyte assembly exhibited similar behavior to CS films on X-ray diffraction patterns. This can be attributed to the fact that the pectin monolayers do not show an independent crystal structure, while the CS monolayer exhibits a semi-crystalline state with weak crystallization [58]. Incomplete crystals with broad and diffuse diffraction peaks could be due to too small grains and defects in the crystals. The worse the crystallinity of the film, the weaker the diffraction ability and the wider the peak shape of the XRD pattern. The bilayer films prepared by polyelectrolyte assembly exhibited similar performance to the CS films, which indicated that the CS layer maintained an independent structure and the pectin layer showed little effect on it. Compared to monolayers, the amplitude of the diffraction peak of the CS-pectin films increased after assembly. The polyelectrolyte assembly promoted bonding between the two substrates, promoting the formation of intermolecular hydrogen bonds and electrostatic interactions. It could be the reason for the variation in the diffraction peaks [59]. The diffraction peaks of the CS-HM films were higher than those of the CS-LM films. Due to the larger molecular weight of HM pectin, the molecular size and viscosity of HM pectin in FFS were higher than that of LM pectin [39,42]. The zeta potential of HM pectin was lower than that of LM pectin. It led to a weaker intermolecular repulsion, resulting in molecular aggregation forming an amorphous structure [60]. The results were in good agreement with the measurements of SEM and WVP.

### 3.9. FT-IR Spectroscopy and the Mechanism of Forming Bilayer Films

**Figure 7:**
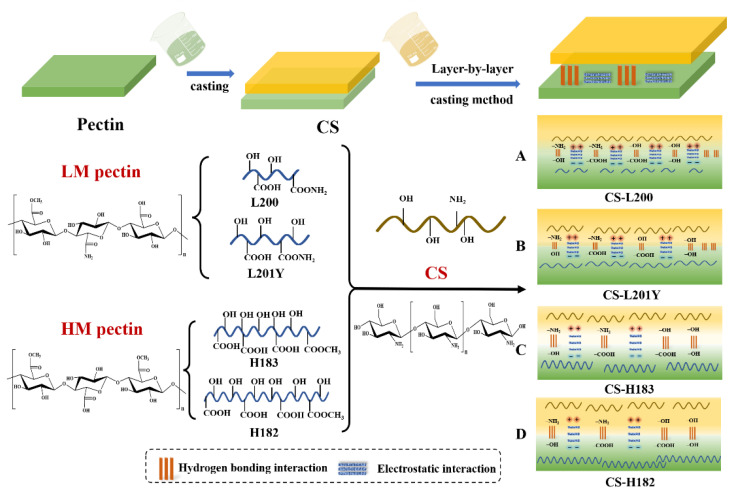
Schematic illustration of polyelectrolyte assembly mechanism among CS and pectin ((**A**): CS-L200, (**B**): CS-L201Y, (**C**): CS-H183, (**D**): CS-H182).

The characteristic regions of FT-IR spectra of the CS, pectin, and CS-pectin films were shown in Figure 6B. For the CS monolayer film, the bands appearing at 3273 and 2929 cm^−1^ correspond to the O–H and C–H stretching vibrations, respectively [61]. At 1635, 1552, and 1330 cm^−1^, it corresponded to the characteristic bands of amide I, amide II, and amide III, respectively [62]. The observable band at 1405 cm^−1^ showed the shear vibration of the –NH_2_ group on the glycosidic bond, and the absorption peak at 1023 cm^−1^ was attributed to the stretching vibration of C–O–C on the glycosidic bond [63]. For the pectin monolayer films, the band at 2929 cm^−1^ reflected the C–H stretching vibration of the CH_2_ group [64]. Due to the absorption of the carboxylic acid group, two vibrations at 1738 cm^−1^ and 1624 cm^−1^ can be observed in the spectrum, corresponding to the absorption of the esterified carboxyl group and the carboxylate ion, respectively [65]. Differences in the absorption peaks of carboxylate ions can be observed in the LM and HM pectin films, and the spectral peaks of the HM pectin film tended to be low-amplitude and broad-band, which reflected more –COOH in the LM pectin. The bands located in the range of 800–1000 cm^−1^ were the absorption peaks of polysaccharide structures such as rhamnose, glucose, etc. [66]. As shown in Figure 6B, no new characteristic peaks appeared in the main bands of the CS-pectin bilayers after polyelectrolyte assembly, which indicated that the interaction between the CS and pectin bilayers was more physical than chemical [67]. Amplitude variation of the spectral peaks can be observed when comparing the monolayer and bilayer films. At 3273 cm^−1^, the absorption peak of the bilayer film appeared red-shifted, and both sides of the bilayer film shifted to a lower wavenumber and lower amplitude compared with the monolayer film. The absorption peak for O–H stretching vibrations (3200–3500 cm^−1^) of the bilayer film was wider than that of the monolayer film, indicating that there was a hydrogen bond interaction between –OH of pectin and –NH_2_ of CS, which reduced the stretching vibration of O–H [34]. As shown in Figure 7, compared with the CS-HM bilayers, the CS-LM films showed stronger hydrogen bonding inside. These phenomena could be ascribed to the following aspects. Firstly, the absorption peak for O–H stretching vibrations of the CS-LM bilayer film was shifted more towards the lower amplitude direction, resulting in a wider absorption peak than that of the CS-HM bilayer film. Secondly, from the SEM results, it also can be observed that the density and compatibility of the LM bilayer film are higher than that of the HM bilayer film. In the bilayer films, the C–O stretching of the ester group at 1738 cm^−1^ of the pectin films disappeared, and the formation of the ester bond between CS and pectin was not detected [68]. In the meanwhile, the absorption at 1330 cm^−1^ of the CS film existed in the bilayer films with no change in amplitude, indicating that there was no chemical reaction between –COOH and –NH_2_ [30,48]. Therefore, the molecular interaction between the CS and pectin layer after bilayer assembly was driven by non-covalent of hydrogen bonds and electrostatic interactions (Figure 7). Furthermore, according to the analysis of DM and zeta potential, it could be observed that the largest force was found between the CS and L200 pectin layer (Figure 7A). The force of polyelectrolyte assembly did not improve the mechanical and barrier properties of the bilayer film by changing the properties of the polymer on the film surface.

From the above characterization results of the physicochemical properties of the films, the CS-L200 bilayer film exhibited a uniform and compatible internal structure of the film without obvious holes and cracks, showing good gas barrier properties and UV shielding properties. Compared with the CS monolayer film, the mechanical properties of the CS-H182 bilayer film were improved by 45.45% (TS) and 41.91% (EAB), respectively, while exhibiting better thermal stability. Therefore, the CS-L200, CS-H182, and their related control films (PE, CS, L200, and H182) were selected for further evaluation of the antifogging property of the polyelectrolyte films.

### 3.10. Antifogging Property

**Figure 8:**
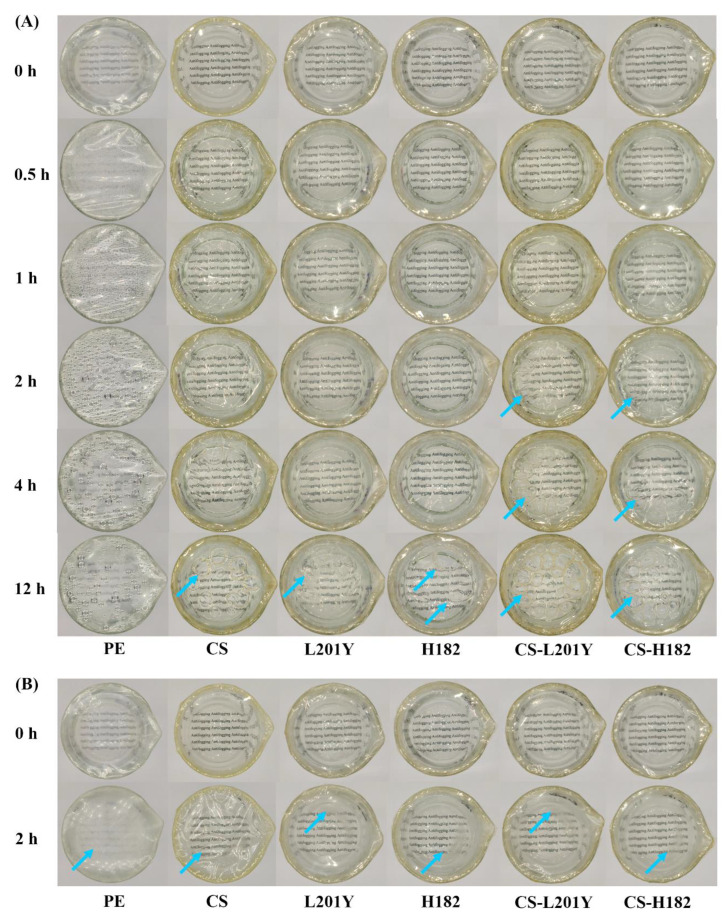
Thermal antifogging property (**A**) and cold antifogging property (**B**) of the monolayer and bilayer films (PE, CS, L201Y, H182, CS-L201Y, CS-H182). The folds and breakage of film were pointed by the blue arrow.

The surface fogging of packaging materials is a common phenomenon. When the ambient temperature changes, water molecules in the air condense on the surface of the packaging material to form water droplets. The antifogging property of packaging materials can ensure good visual effects and reduce the growth of microorganisms [69]. The results of the thermal antifogging experiment were shown in Figure 8A. At the initial time, all the films were in a transparent state, and the text in the background was clearly visible. When the film-coated beaker was transferred from a room-temperature environment (25 °C, 50% RH) to a 50 °C water bath, different phenomena were observed. After being placed in a 50 °C water bath for half an hour, a large number of tiny water droplets appeared on the inside of the PE film, but no water droplets were formed in the five polysaccharide films, indicating that the CS film, pectin film, and CS-pectin bilayer films show a certain water absorption capacity and antifogging properties. After 2 h in the water bath, a large number of water droplets appeared on the inner side of the PE film, and tiny water droplets appeared on the inner surface of the L200 pectin film and H182 pectin film. It showed a small amount of swelling and no water droplet formation in the CS film, CS-L200, and CS-H182 bilayer films. After being placed in a 50 °C water bath for 12 h, larger water droplets appeared in the L200 pectin film, while larger holes appeared in the H182 pectin film. The CS and polyelectrolyte bilayer film showed swelling phenomena, and the swelling phenomenon of the CS-H182 bilayer film was smaller than that of the CS-L200 bilayer film. The above results were consistent with the WVP results as shown in Table 3. When a large number of water droplets appear in the PE film, the water vapor could not pass through the PE film. The antifogging effect of the CS film was better than that of the pectin film, which could be attributed to the higher WVP value of CS. The excellent antifogging effect of the polyelectrolyte bilayer films was caused by the good absorption and transport ability of water molecules by the hydrophilic groups of CS and pectin [70,71]. The water molecules in the condensed water droplets were rapidly absorbed into the hydrophilic regions of the film components through hydrogen bonding and ionic dipole interactions [72]. There were differences in the antifogging effect of the bilayer films. Among them, the L200 pectin component in the CS-L200 bilayer film showed a lower DM, and more hydrophilic groups in the component could uniformly disperse more water molecules in the film through hydrogen bonding. This led to a more obvious swelling phenomenon of the attached CS components. These results showed that the CS-H182 bilayer film showed better antifogging properties. The results of the cold antifogging test were shown in Figure 8B. The film-coated beaker was transferred from a −20 °C environment to a room-temperature environment (25 °C, 50% RH). The surface of the PE film was rapidly atomized, the CS and pectin monolayer films were atomized in a certain range, and the surface of the bilayer films was slightly atomized. This was attributed to the fact that the bilayer films contain more hydrophilic groups to generate a strong affinity for water molecules, and the bilayer film components produce stronger water molecule retention ability. For food packaging, fogging and condensation on the surface of the packaging material hinder the observation of the freshness and color of the food, and the deposition of water droplets on the food packaging substrate can lead to bacterial growth and food spoilage. Antifogging experiments showed that the CS-H182 polyelectrolyte bilayer films had the function of preventing water vapor from being atomized inside or on the surface of the film and showed fresh-keeping application value.

## 4. Conclusions

In this study, the CS-pectin polyelectrolyte bilayer films were successfully prepared by the LBL casting method using CS and four types of citrus pectin as film-forming substrates. The FT-IR, XRD, SEM, and AFM analysis showed that there are hydrogen bonds and electrostatic interactions between the CS and the pectin layer. Indeed, the interactions between CS and pectin were closely related to the DM, *M*_w_, and zeta potential of pectin. In combination with the low DM and *M*_w_ pectin, the CS-pectin film formed excellent properties of UV shielding and gas barrier. Meanwhile, the bilayer film composed of the high DM and *M*_w_ pectin exhibited outstanding compression resistance and antifogging properties. Therefore, polyelectrolyte bilayer films with specified functionality could be prepared by combining citrus pectin structural diversity with CS. The eco-friendly polyelectrolyte bilayer film was expected to be used in the production of materials for packaging fruits and vegetables and replace a large number of current non-renewable plastic materials.

## Figures and Tables

**Table 1:** Degree of esterification and amidation, weight average molecular weights, and number average molecular weights values of the CS and pectin.

	DE (%)	DA (%)	*M*_w_ (kDa)	*M*_n_ (kDa)	*M*_w_/*M*_n_
CS	−	−	619.5	514	1.21
L200	26.9	21.9	87.46	16.4	5.33
L201Y	26.5	23.7	369.84	27.1	13.64
H183	68.9	−	873.34	54.1	16.15
H182	69.5	−	1243.64	53.3	23.31

**Table 2:** Color parameters (*L*, *a*, *b*, Δ*E*) and opacity of different monolayer and bilayer films.

Film	Color					Opacity (mm^–1^)
	*L*	*a*	*b*	∆E	WI	
CS	37.79 ± 0.16 ^a^	–0.36 ± 0.23 ^a^	–0.45 ± 0.40 ^ab^	56.06 ± 0.14 ^e^	37.78 ± 0.16 ^a^	1.19 ± 0.07 ^a^
L200	35.17 ± 0.62 ^cd^	–0.40 ± 0.05 ^a^	–0.33 ± 0.16 ^ab^	58.42 ± 0.61 ^abc^	35.16 ± 0.62 ^cd^	1.12 ± 0.10 ^a^
L201Y	34.42 ± 0.14 ^e^	–0.31 ± 0.06 ^a^	–0.01 ± 0.17 ^a^	59.16 ± 0.14 ^a^	34.42 ± 0.14 ^e^	1.12 ± 0.03 ^a^
H183	34.96 ± 0.43 ^de^	–0.38 ± 0.07 ^a^	–0.48 ± 0.05 ^ab^	58.63 ± 0.43 ^ab^	34.96 ± 0.43 ^de^	1.14 ± 0.42 ^a^
H182	34.65 ± 0.18 ^de^	–0.30 ± 0.09 ^a^	–1.11 ± 1.10 ^b^	58.98 ± 0.12 ^ab^	34.63 ± 0.16 ^de^	1.16 ± 0.06 ^a^
CS-L200	35.73 ± 0.00 ^bc^	–0.45 ± 0.01 ^a^	–0.33 ± 0.04 ^ab^	57.86 ± 0.01 ^cd^	35.73 ± 0.00 ^bc^	1.03 ± 0.05 ^a^
CS-L201Y	35.98 ± 0.15 ^b^	–0.46 ± 0.03 ^a^	–0.35 ± 0.12 ^ab^	57.61 ± 0.15 ^d^	35.98 ± 0.15 ^b^	1.01 ± 0.03 ^a^
CS-H183	35.35 ± 0.25 ^bcd^	–0.40 ± 0.03 ^a^	–0.23 ± 0.30 ^a^	58.23 ± 0.24 ^bcd^	35.35 ± 0.25 ^bcd^	0.99 ± 0.04 ^a^
CS-H182	34.94 ± 0.76 ^de^	–0.38 ± 0.07 ^a^	–0.16 ± 0.02 ^a^	58.64 ± 0.76 ^ab^	34.94 ± 0.76 ^de^	0.97 ± 0.04 ^a^

Values represent the mean ± standard deviation. Different letters within the same column indicate significant differences (*p* < 0.05) in value by using Duncan’s test.

**Table 3:** Moisture content, water solubility, and barrier properties of different monolayer and bilayer films.

Film	MC (%)	WS (%)	WVP × 10^10^ (g·m^–1^·s^–1^·Pa^–1^)	OP × 10^9^ (g·m^–1^·s^–1^)	CDP × 10^9^ (g·m^–1^·s^–1^)	CDP/OP
CS	19.81 ± 0.85 ^a^	13.73 ± 0.13 ^c^	1.46 ± 0.11 ^a^	1.39 ± 0.02 ^a^	2.75 ± 0.34 ^abc^	1.98
L200	10.95 ± 0.07 ^b^	74.70 ± 7.91 ^a^	0.99 ± 0.07 ^d^	1.14 ± 0.12 ^ab^	3.04 ± 0.59 ^ab^	2.67
L201Y	12.50 ± 2.21 ^b^	74.89 ± 1.47 ^a^	0.99 ± 0.09 ^d^	1.00 ± 0.22 ^bc^	3.12 ± 0.30 ^a^	3.12
H183	11.30 ± 0.44 ^b^	73.41 ± 3.64 ^a^	1.06 ± 0.11 ^d^	0.96 ± 0.35 ^bc^	2.99 ± 0.52 ^ab^	3.11
H182	12.89 ± 4.64 ^b^	75.88 ± 2.27 ^a^	1.10 ± 0.05 ^cd^	0.95 ± 0.20 ^bc^	2.94 ± 0.13 ^ab^	3.09
CS-L200	12.46 ± 0.12 ^b^	39.67 ± 0.85 ^b^	1.16 ± 0.05 ^bcd^	0.75 ± 0.09 ^c^	2.24 ± 0.35 ^c^	2.99
CS-L201Y	13.38 ± 2.64 ^b^	40.11 ± 1.15 ^b^	1.12 ± 0.07 ^bcd^	0.73 ± 0.07 ^c^	2.22 ± 0.15 ^c^	3.04
CS-H183	12.20 ± 0.68 ^b^	40.22 ± 1.72 ^b^	1.24 ± 0.10 ^bc^	0.71 ± 0.05 ^c^	2.38 ± 0.35 ^bc^	3.35
CS-H182	13.66 ± 2.30 ^b^	41.65 ± 0.90 ^b^	1.28 ± 0.14 ^b^	0.70 ± 0.07 ^c^	2.40 ± 0.20 ^bc^	3.43

Values represent the mean ± standard deviation. Different letters within the same column indicate significant differences (*p* < 0.05) of value by using Duncan’s test.

**Table 4:** Thickness and mechanical properties of different monolayer and bilayer films.

Film	Film Thickness (mm)	TS (MPa)	EAB (%)
CS	0.082 ± 0.002 ^a^	49.15 ± 6.14 ^b^	4.70 ± 0.48 ^b^
L200	0.062 ± 0.004 ^e^	21.15 ± 2.68 ^d^	2.59 ± 0.74 ^c^
L201Y	0.063 ± 0.004 ^de^	23.86 ± 3.42 ^cd^	3.88 ± 0.57 ^bc^
H183	0.065 ± 0.003 ^cde^	24.64 ± 2.38 ^cd^	3.96 ± 1.19 ^bc^
H182	0.070 ± 0.002 ^c^	30.61 ± 8.62 ^c^	3.93 ± 1.58 ^bc^
CS-L200	0.065 ± 0.005 ^cde^	47.62 ± 0.50 ^b^	3.22 ± 0.58 ^bc^
CS-L201Y	0.066 ± 0.001 ^cde^	50.89 ± 1.74 ^b^	4.55 ± 0.47 ^b^
CS-H183	0.067 ± 0.003 ^cd^	68.46 ± 6.16 ^a^	6.31 ± 0.12 ^a^
CS-H182	0.075 ± 0.002 ^b^	71.49 ± 1.89 ^a^	6.67 ± 1.18 ^a^

Values represent the mean ± standard deviation. Different letters within the same column indicate significant differences (*p* < 0.05) in value by using Duncan’s test.

## Data Availability

The data presented in this study are available from the corresponding author upon request.
